# Effect of Pt Decoration on the Optical Properties of Pristine and Defective MoS_2_: An Ab-Initio Study

**DOI:** 10.3390/ijms231911199

**Published:** 2022-09-23

**Authors:** Juan Manuel Ramírez-de-Arellano, Ali Fransuani Jiménez-González, Mónica Canales, Luis Fernando Magaña

**Affiliations:** 1 Tecnologico de Monterrey, Escuela de Ingeniería y Ciencias, Calle del Puente 222, Mexico City 14380, Mexico; 2 Instituto de Física, Universidad Nacional Autónoma de México, Apartado Postal 20-364, Mexico City 01000, Mexico

**Keywords:** ab-initio, DFT calculations, 2D materials, MoS_2_, optical properties, platinum, FPMD

## Abstract

Using structural relaxation calculations and first-principles molecular dynamics (FPMD), we performed numerical simulations to explore the interaction of a 2D MoS_2_ surface and a platinum atom, calculating the optical properties of the resulting material. We explored three initial positions for the interaction of the Pt atom and the pristine MoS_2_ surface, plus another position between Pt and the MoS_2_ surface with a sulfur vacancy V_S_. The surface absorbed the Pt atom in all cases considered, with absorption energies ranging from −2.77 eV to −5.83 eV. We calculated the optical properties and band structure of the two cases with the largest absorption energies (−3.45 eV and −5.83 eV). The pristine MoS_2_ is a semiconductor with a gap of around 1.80 eV. With the adsorption of the Pt atom (the −3.45 eV case), the material reduces its band gap to 0.95 eV. Additionally, the optical absorption in the visible range is greatly increased. The energy band structure of the 2D MoS_2_ with a sulfur vacancy V_S_ shows a band gap of 0.74 eV, with consequent changes in its optical properties. After the adsorption of Pt atoms in the V_S_ vacancy, the material has a band gap of 1.06 eV. In this case, the optical absorption in the visible range increases by about eight times. The reflectivity in the infrared range gets roughly doubled for both situations of the Pt-absorbed atom considered. Finally, we performed two FPMD runs at 300 K to test the stability of the cases with the lowest and highest absorption energies observed, confirming the qualitative results obtained with the structural relaxations.

## 1. Introduction

Bidimensional materials show different interesting physical properties, making them suitable for many potential applications, including energy storage [1,2,3], biomedical research [4,5,6], field-effect transistors (FETs) [7,8,9,10], as well as sensors and biosensing [11,12]. One of these materials is Molybdenum disulfide, MoS_2_, a layered dichalcogenide with a hexagonal structure reminiscent of graphene. Like graphene, the bonds between layers are weaker, allowing for a relatively easy dislocation [13,14,15,16]. Monolayer MoS_2_ is also a direct-gap semiconductor with a band gap of 1.8 eV [17] with potential applications that have been explored in fields as diverse as ultrafast photonics, the treatment of antibiotic-polluted water, drug-delivery purposes, water splitting, and FETs [18,19,20,21,22,23,24].

Studying the band structure and optical properties of such 2D materials helps explore their potential applications. The band structure of MoS_2_ has been previously explored, including the effect of interlayer pressure [25]. Here, we studied the changes produced in the optical properties of MoS_2_ when adsorbing Pt. The present work consists of two stages: First we performed static calculations on four cases of interaction (see Section 2.2). Then, in a second stage, we took two cases—the lowest and the highest adsorption energies—and calculated their optical properties and band structure (Section 2.3 onwards). These results were compared with those of the pristine MoS_2_ surface. Additionally, we performed first principles molecular dynamics (FPMD) calculations at 300 K on two cases to further explore the qualitative behavior that was previously found with the structural relaxations.

In this work we considered pristine MoS_2_ as well as MoS_2_ with a sulfur monovacancy (also labeled as Vs) on the unit cell. Vacancies on MoS_2_ have been previously studied experimentally and by first-principles calculations, finding relatively low formation energies for a Vs vacancy [16,26], which in turn makes it relatively easy to find.

## 2. Results

### 2.1. Pristine MoS_2_ Layer

**Figure 1:**
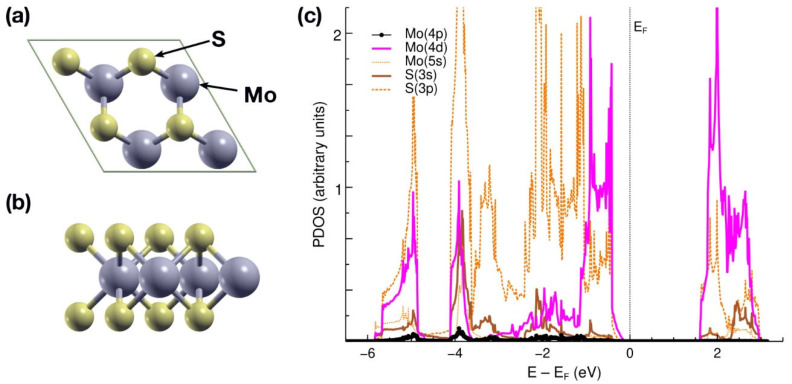
(**a**) XY-plane view of the unit cell considered for the pristine MoS_2_. It contains 12 atoms: four Mo and eight S atoms. The cell parameter is 6.3 Å. (**b**) XZ-plane view of the unit cell. (**c**) The PDOS for the pristine MoS_2_, showing a gap of 1.8 eV.

Figure 1a,b shows the hexagonal unit cell considered. With a cell parameter of 6.3 Å, it contains 12 atoms: eight S atoms and four Mo atoms. We chose the size of the cell to ensure a sufficiently long distance between one element of the system and its repetition in the next cell since the code we used (Quantum ESPRESSO) considers periodic boundary conditions. The unit cell is then large enough to give a sufficiently good amount of information while avoiding spurious interactions. Starting from this cell, we removed one S atom to create a vacancy. After performing a structural relaxation on the pristine MoS_2_, we calculated its projected density of states (PDOS) [27], obtaining a gap of 1.8 eV (see Figure 1c), which is in agreement with previously reported works [25]. Below the Fermi level, there is a hybridization of orbitals p and d from molybdenum with orbitals s and p from sulfur. Above 2 eV, there is a hybridization of the same orbitals, but the contribution of orbital p from Mo is negligible.

### 2.2. Static Calculations: Pt-Absorption on the MoS_2_ Layer

**Figure 2:**
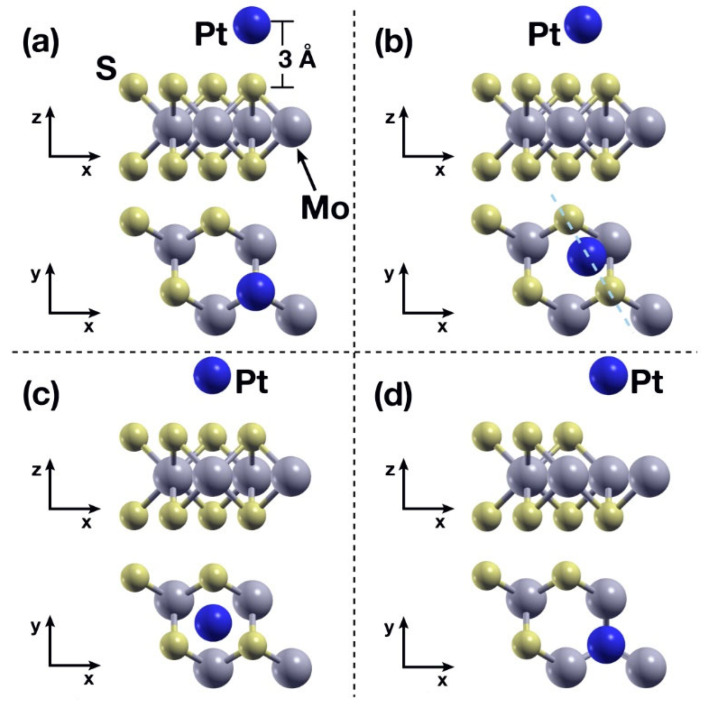
Initial configurations considered for the static calculations. The Pt atom is placed (**a**) above an S atom; (**b**) at the midpoint above the line between two adjacent S atoms; (**c**) above the center of the triangle formed by three adjacent S atoms; (**d**) Above a previously introduced V_S_ vacancy. In all cases, the vertical distance between the Pt atom and the plane of S atoms was 3 Å.

We considered four initial configurations for the interaction between the Pt atom and the MoS_2_ surface, as shown in Figure 2. The Pt atom was placed in the following initial positions: Directly above an S atom (Figure 2a); directly above the bonding line between two adjacent S atoms (Figure 2b); above the center of the triangle formed by three adjacent S atoms (Figure 2c); and above a V_S_ vacancy (Figure 2d). In the last case, the system was previously relaxed structurally so the V_S_ vacancy would be properly taken into account. In all cases, the initial vertical distance between the Pt atom and the superior plane of S atoms was 3 Å. 

**Figure 3:**
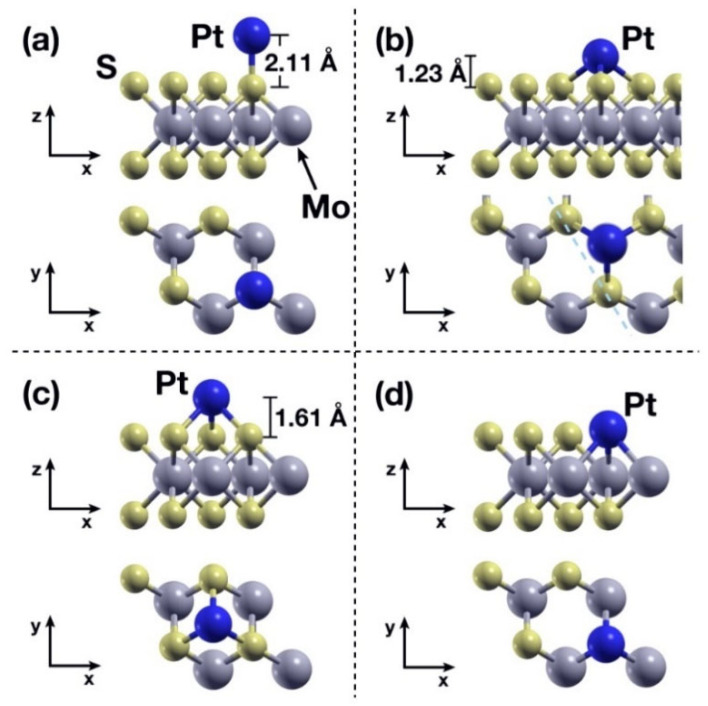
Final configurations considered for the static calculations of Section 2.2. The Pt atom is initially placed (**a**) above an S atom; (**b**) at the midpoint above the line between two adjacent S atoms; (**c**) above the center of the triangle formed by three adjacent S atoms; (**d**) Above a previously introduced V_S_ vacancy. In the four cases the absorption energies can be catalogued as chemisorption [28], with the largest energy being that of case (**d**): The Pt atom is absorbed by the surface at the V_S_ vacancy site. See also Table 1.

Figure 3 shows the final configurations of the corresponding cases from Figure 2, while Table 1 shows the absorption energies for each case, obtained according to Equation (5), Section 4. In case (b), the Pt atom displaces horizontally as well, ending up directly above the closest Mo atom (Figure 3b). In Figure 3b, we included part of the repeated cell—due to the use of periodic boundary conditions—to show that the Pt atom is anchored by the closest three S atoms and the Mo atom directly below it, resulting in this case being the second strongest of the four considered. Case (d)—the Pt atom being absorbed in the V_S_ site—is the one with the strongest chemisorption interaction (Figure 3d). 

### 2.3. PDOS for the Pristine and Defective MoS_2_ Surfaces + Pt

Of the four cases considered above, we calculated the PDOS, band structures and optical properties for cases (b) and (d) from Figure 2 and Figure 3, as they were the ones with the strongest interactions. We took only these two cases to keep this work from being unnecessarily large, while still being able to extract significant conclusions from the results. In particular, the largest absorption energy of case (b)—when compared with the other two cases (a and c) involving a Pt atom and the pristine surface—would also make it the most stable and likely among cases a, b and c.

**Figure 4:**
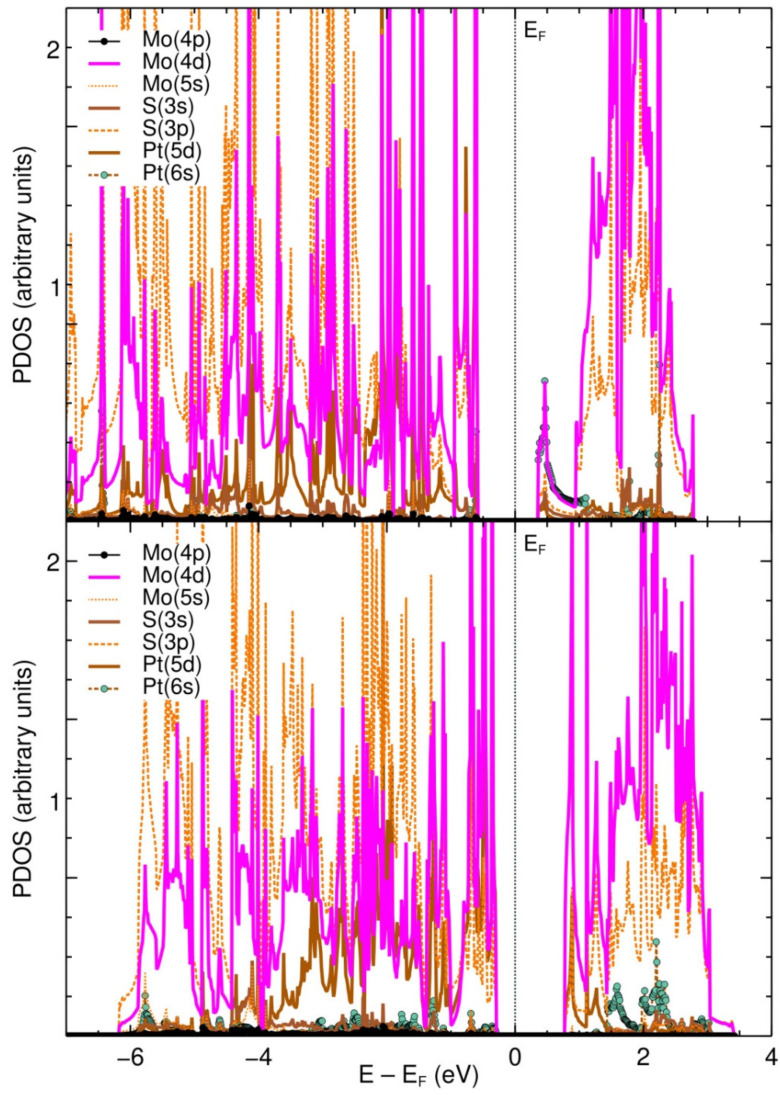
(**Top**) The PDOS of the pristine MoS_2_ interacting with a Pt atom, corresponding to case (b) in Figure 2 and Figure 3. Here, the Pt atom was initially placed above an S-S bond. The band gap is reduced to 0.95 eV in this case. (**Bottom**) The PDOS of the MoS_2_ + V_S_ system after interacting with a Pt atom, corresponding to case (d) in Figure 2 and Figure 3. The Pt atom was initially placed above the V_S_ vacancy, this being the strongest interaction among the cases considered. The band gap is 1.06 eV in this case.

Figure 4 (top) shows the PDOS of the resulting optimized configuration (See Figure 3b). The effect of the absorbed Pt atom is a reduction of the band gap, which for this case was found to be 0.95 eV. Between −7.0 and 0 eV, there is a strong hybridization between the Mo 4d orbital, the Pt 5d orbital and the S 3s and 3p orbitals. A weaker hybridization with the 4p Mo orbital is also present. Between 1.0 and 3.5 eV there is a strong hybridization as well, this time between the Mo 4d orbital, the S 3s and 3p orbitals, and the Pt 6s orbital.

Figure 4 (bottom) shows the PDOS for case (d), in which the Pt atom is absorbed on the V_S_ site of the MoS_2_ + V_S_ system. The band gap reduction—from 1.8 eV to 1.06 eV—is not as big as in case (b), when compared to the pristine MoS_2_ surface. The orbitals hybridization is overall similar to the previous case.

### 2.4. Band Structures of the Pristine and Defective MoS_2_ Surfaces + Pt

**Figure 5:**
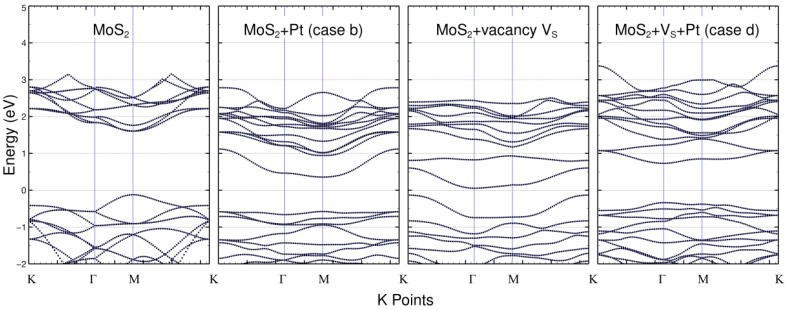
The energy band structure calculations for the pristine MoS_2_, the same system with an adsorbed Pt atom, the surface with vacancies, and the latter with an adsorbed Pt atom. The Fermi energy is normalized at 0 eV. The band gaps are1.8 eV, 0.95 eV, 0.74 eV and 1.06 eV, respectively.

Using the final relaxed configurations of the pristine case plus the cases considered in Section 2.2 and Section 2.3, as well as the MoS_2_ + V_S_ surface alone, we calculated the energy band structure of each case. In Figure 5, showing the results, the Fermi energy is normalized at zero.

The proposed manipulation of the MoS_2_ surface causes an overall reduction in the band gap related to the pristine surface. The pristine surface is a semiconductor with a band gap of 1.8 eV, as expected. The adsorption of a Pt atom on the pristine MoS_2_ (case (b)) does not change that property, but it reduces the band gap to 0.95 eV. Interestingly, the addition of a V_S_ vacancy induces a further reduction in the band gap, down to 0.74 eV. But this change gets overturned by the adsorption of a Pt atom on the V_S_ site, and the band gap gets increased to 1.06 eV. The changes in the band structure implied substantial changes in the optical properties of the surface, as shown in the next section.

### 2.5. Optical Properties

**Figure 6:**
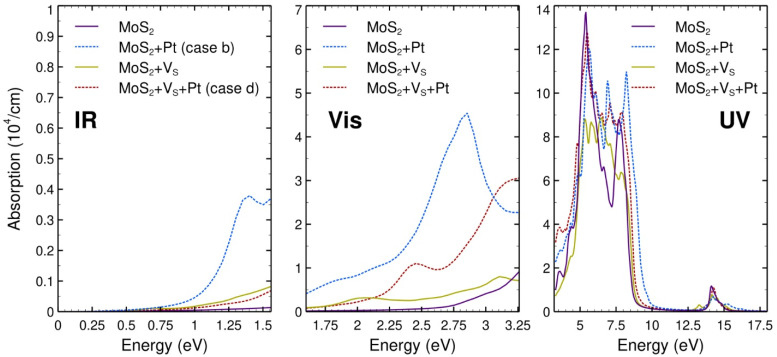
Absorption spectra in the infrared (**left**), visible (**center**), and UV (**right**) ranges, along the direction perpendicular to the surface for the systems we have investigated: pristine MoS_2_; Pt adsorbed on pristine MoS_2_ (case b); MoS_2_ with a sulfur vacancy (MoS_2_ + V_S_); and the latter surface with a Pt atom adsorbed on the V_S_ site (MoS_2_ + V_S_+Pt). The vertical scale is the same for the three sections of the plot, but the range shown differs to make the features of each section more clear.

**Figure 7:**
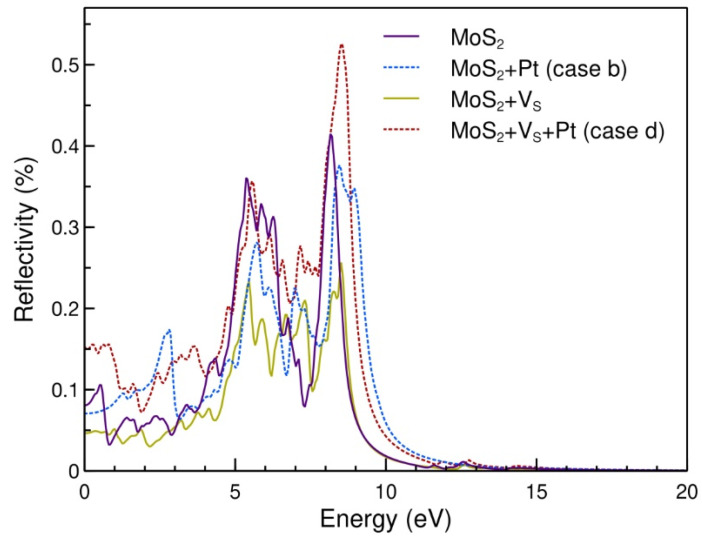
Reflectivity spectra for the cases considered. The substitutionally absorbed Pt atom increases the reflectivity overall. Most of the reflectivity is observed in the visible spectra, with a negligible amount observed at energies beyond 20 eV.

For the cases considered from Section 2.2 onwards, we calculated the optical absorption spectra in the infrared (IR), visible (VIS), and ultraviolet (UV) range along the Z-axis (see Figure 6). Figure 7 shows the reflectivity.

The optical absorption in the infrared region has its most significant values for the surface of Pt adsorbed on pristine MoS_2_ and the smallest for MoS_2_ with a vacancy. We have the same behavior in the visible range, except for the interval between 2.90 eV and 3.25 eV, where the smallest values correspond to MoS_2_ with a vacancy. In the ultraviolet region, Pt on the pristine MoS_2_ has the most significant absorption, keeping the overall shape related to pristine MoS_2_ with about the same positions for peaks and valleys. In this case, the absorption between 6.00 eV and 7.00 eV is approximately 46% larger compared to the pristine surface.

In the case of reflectivity, the most substantial change related to pristine MoS_2_ is between 0 eV and 5.00 eV and comes from the Pt adsorbed on non-defective MoS_2_. In the same region, the smallest values correspond to MoS_2_ with a vacancy. The most considerable value for reflectivity is for Pt adsorbed on defective MoS_2_, which occurs around 8.6 eV (see Figure 7).

### 2.6. FPMD Calculation for the Weakest and Strongest Interactions

**Figure 8:**
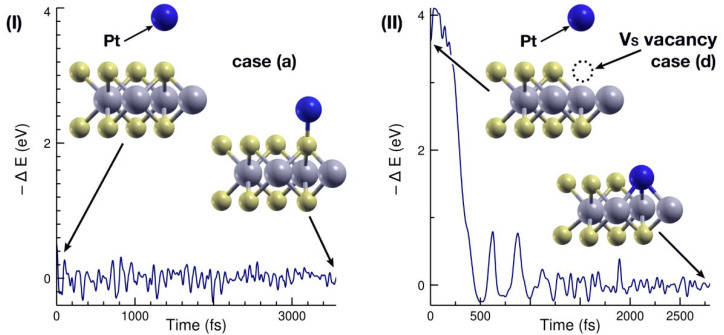
Energy evolution for the first principles molecular dynamics calculations performed. We considered the two extreme cases: (**I**) those with the lowest absorption energy, corresponding to case (a) in Figure 2 and Figure 3; and (**II**) with the highest one, corresponding to case (d) in Figure 2 and Figure 3. The FPMD calculations shown ran for 3561 and 2800 iterations, respectively. In both cases the qualitatively behavior agrees with the structural relaxation calculations previously performed.

Finally, to explore the stability of the combined systems in real-life situations, we used first principles molecular dynamics calculations (see the Materials and Methods section for more details on the FPMD calculation). We chose the weakest and strongest interaction energies—cases (a) and (d) from Figure 2 and Figure 3—for an FPMD calculation at 300 K. Figure 8 shows the initial and final configurations of said cases, along with an energy evolution plot. In both cases, the initial configuration was the same as that considered in the structural relaxations.

The ab initio molecular dynamics calculation at 300 K showed fundamentally the same qualitative behavior, with the MoS_2_ surface absorbing the Pt atom. Figure 8I shows the initial and final positions of the Pt atom during the adsorption process on the pristine MoS_2_ layer (case b), along with the energy evolution of the system during the 3561-iterations FPMD calculation.

Figure 8II shows a similar plot for the FPMD calculation of the Pt interacting with the MoS_2_ + V_S_ system. Again, the qualitative behavior of the system is the same as that of Section 2.2. The FPMD run consisted of 2800 iterations and the Pt atom is absorbed in the V_S_ site rather early in the calculation.

## 3. Discussion

We performed static calculations and then FPMD simulations to investigate the Pt adsorption effect on the optical properties of 2D MoS_2_. We considered pristine and defective MoS_2_ at 300 K and atmospheric pressure. The initial static calculations show that the strongest interaction (chemisorption) occurs when the Pt atom is absorbed at a V_S_ site of the surface, with an absorption energy of −5.83 eV. When a pristine MoS_2_ surface is considered, the strongest interaction occurs when the Pt atom is initially placed above an SS bond line, with it ending up being absorbed above the closest Mo atom.

The inclusion of either the Pt atom or a V_S_ vacancy on the MoS_2_ surface results in an overall reduction of its band gap. The initial pristine surface is found to be a semiconductor with a band gap of 1.8 eV, which agrees with previous works. The adsorption of a Pt atom (case b) reduces the band gap to 0.95 eV. Including a V_S_ vacancy reduces the band gap to 0.74 eV, as seen from the band structure diagram (Figure 5). However, the subsequent adsorption of a Pt atom on the V_S_ site (case d) again increases the band gap to a value of 1.06 eV. The changes in the band structure implied substantial changes in the optical properties of the surface. It remains a point of interest to use the Kubelka–Munk function relation along with its Tauc plot [29] to confirm the band-gap values obtained, considering that the method is robust mainly for polycrystalline semiconductors [30].

Regarding previous experimental and theoretical results on the interaction between Pt and MoS_2_, it is known that Pt atoms are more likely to occupy sites on a tubular MoS_2_ structure rather than on a planar one [31]. On the planar 2D MoS_2_, the Pt atoms tend to cluster. Thus, the Pt decoration for photocatalysis or gas sensors on 2D MoS_2_ involves Pt nanoparticles [32,33,34,35] instead of single Pt atoms. Our results may explain this fact. The cohesive energy of Pt is 5.84 eV/atom [36], while we obtained an adsorption energy of 3.45 eV for a Pt atom on the pristine 2D MoS_2_ surface. It can be inferred then that the Pt atoms will tend to cluster instead of being adsorbed on the surface. Furthermore, we found that the adsorption energy of a Pt atom on a V_S_ vacancy (5.83 eV) is strikingly close to the Pt cohesive energy. In this way, a 2D MoS_2_ surface with V_S_ vacancies could likely absorb a single Pt atom on the vacancy.

A question arises about the possibility of plasmons causing an enhanced Pt decoration on the MoS_2_ surface, as plasmons are present in metallic systems. When MoS_2_ is decorated with Pt nanoparticles—which are small metallic particles—we would have plasmons on those particles. But in the system considered in this work, we included a Pt atom, not metallic nanoparticles decorating the surface. Thus, we don’t expect to have plasmons.

The vacancy we are considering in our unit cell is equivalent to a 12.5% vacancy density on the surface. For future works it is of interest to explore the effects that varying the vacancy density could have on the MoS_2_ properties and on its interaction with Pt.

The optical absorption in the infrared region has its most significant values for the surface of Pt adsorbed on pristine MoS_2_ (case b) and the smallest for MoS_2_ with a vacancy. We have the same behavior in the visible range, except for the interval between 2.90 eV and 3.25 eV, where the smallest values correspond to MoS_2_ with a vacancy. In the ultraviolet region, Pt on the pristine MoS_2_ has the most significant absorption, keeping the overall shape related to pristine MoS_2_ with about the same positions for peaks and valleys. In this case, the absorption between 6.0 eV and 7.0 eV is approximately 46% larger compared to the clean material.

For the reflectivity, the most substantial change related to pristine MoS_2_ is between 0 eV and 5.00 eV and comes from the Pt adsorbed on non-defective MoS_2_. In the same region, the smallest values correspond to MoS_2_ with a vacancy. The maximum reflectivity is for Pt adsorbed on defective MoS_2_, which occurs at around 8.60 eV.

Understanding the optical properties of MoS_2_—and the effect that vacancies alone and in combination with the Pt decoration have on them—is essential, as it could be helpful in the developing of FETs-related technologies. The modulation of the band structure and its related optical properties found in this work could be extended to other surfaces of the transition metal dichalcogenides such as tungsten disulfide or WS_2_. This material has a similar 2H phase structure and a direct band gap as MoS_2_ [16,37], but more research is needed on it to explore the transferability of these results to it and to other 2D materials of the same family.

For future research we are exploring the potential of these combined systems in sensor technologies, particularly for pollutant molecule sensing devices.

## 4. Materials and Methods

All ab initio calculations in this work were made using the Quantum ESPRESSO code [38,39] within the Density Functional Theory (DFT), the pseudopotential formalism, and the projector-augmented wave (PAW) method [40]. All the calculations were non-relativistic, non-spin polarized, with cut-off energy of 80 Ry (1088 eV), and threshold energy for convergence of 1.0 × 10^–6^ eV. This code suite considers periodic boundary conditions and plane-wave expansions. For the static calculations we considered an 8 × 8 × 2 k-mesh grid, using the PBE XC functional expression [41] and the semiempirical Grimme’s DFT-D3 Van der Waals correction [42]. The terms “structural relaxations” and “static calculations” are used indistinctly in this work and correspond to the calculation option ‘relax’ in the Quantum ESPRESSO input file.

Additionally, we considered Born-Oppenheimer first principles molecular dynamics (FPMD) as implemented by Quantum ESPRESSO. FPMD is the method chosen whenever bonds may be broken or formed, or in the presence of complex bonding as in transition metals, which is the case here. We were also interested in considering the effect of vibrations, rotations, velocities, and interactions for all particles of our system, and FPMD allows us to do that. Due to its nature, large and “chemically complex” systems can be better handled by FPMD methods [27,43,44,45]. The FPMD calculations considered a 551 k-points mesh within the Monkhorst–Pack special k-point scheme [46], which is the same scheme considered in the static calculations. Stochastic velocity rescaling is used to control the temperature of 300 K considered in this work. For the time step, we used the default value of 20.0 a.u. in Rydberg atomic units (not Hartree atomic units), where 1 a.u.= 4.8378 × 10^−17^ s = 0.048378 fs. This is equivalent to a time step of 0.96756 ≈ 1 fs. The convergence parameter considered for the MD calculations was set as 1.0 × 10^–4^ eV.

The valence electronic states considered are, for hydrogen: 1s, for molybdenum: 4d^5^ 5s^1^, for sulfur: 3s^2^ 3p^4^, for platinum: 5d^9^ 6s^1^. As a previous step to calculate the PDOS using the projwfc.x program of Quantum ESPRESSO, we performed geometrical optimizations with an 882 k-points mesh. XCrySDen software was used for visualization purposes [47].

We calculated the energy band structure to obtain the imaginary part of the dielectric tensor. We used the Kramers-Kronig relations [48] to obtain the real part, following the procedure explained in more detail in a previous work [49]. We obtained the reflectivity and the optical absorption by considering the two components of the tensor, using the following equations (where *n* is the refractive index and *k* is the extinction coefficient):

(1) $$R_{ii}\left(\omega \right)=\frac{{\left(n-1\right)}^{2}{+k}^{2}}{{\left(n+1\right)}^{2}{+k}^{2}}$$

(2) $$A_{ii}\left(\omega \right)=\frac{2\omega k\left(\omega \right)}{c}$$

where

(3) nii=|εii(ω)|+Reεii(ω)2 

(4) kii(ω)=|εii(ω)|− Reεii(ω)2

We calculated the adsorption energy for all static calculation cases, using the following formula [28]:

(5) $$E_{\mathrm{adsorption}}{=E}_{\mathrm{system}\;1+\mathrm{system}\;2}-E_{\mathrm{system}\;1}-E_{\mathrm{system}\;2}$$

Each term of the right side in Equation (5) is taken from the converged structural relaxation calculation of the corresponding system.

## Figures and Tables

**Table 1:** Adsorption energies E_ads_ (in eV) of the Pt atom on the MoS_2_ surface, for the cases considered in static calculations. The energies are calculated according to Equation (5) from Section 4.

Case ^1^	Description	E_ads_
a	Pt over S, pristine MoS_2_	−2.77
b	Pt over S-S bond, pristine MoS_2_	−3.45
c	Pt over S-S-S triangle, pristine MoS_2_	−2.94
d	Pt over V_S_ vacancy	−5.83

^1^ The labelling of the cases corresponds to that in Figure 2 and Figure 3.
